# Supplementary material for: A proximity ligation screen identifies SNAT2 as a novel target of the MARCH1 E3 ubiquitin ligase
Source: Biol Open. 2026 Aug 7;15(8):bio061700. doi: 10.1242/bio.061700 (PMC13488409; doi:10.1242/bio.061700)
Supplement: Supplementary file 1 [file biolopen-15-061700-s1.pdf]

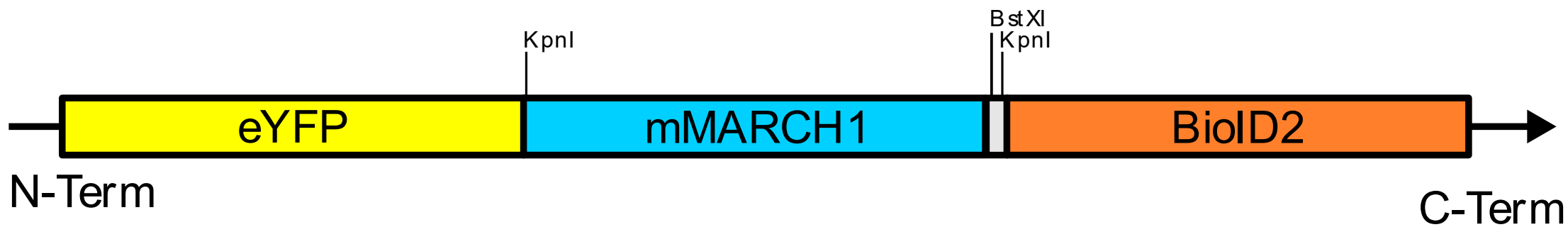

GCGGCCGCGCCACCATGGTTTCTAAAGGCGAGGAACTGTTCACCGGCGTGGTGCCTATTCTG
GTGGAACTGGATGGCGACGTGAACGGCCACAAGTTCTCCGTTTCTGGCGAAGGCGAAGGGG
ACGCCACATACGGAAAGCTGACCCTGAAGTTCATCTGCACCACCGGCAAGCTGCCTGTGCCT
TGGCCTACACTGGTCACCACATTTGGCTACGGCCTGCAGTGCTTCGCTAGATACCCCGACCAT
ATGAAGCTGCACGACTTCTTCAAGAGCGCCATGCCTGAGGGCTACGTGCAAGAGAGAACCAT
CTTCTTTAAGGACGACGGCAACTACAAGACCAGGGCCGAAGTGAAGTTCGAGGGCGACACC
CTGGTCAACAGAATCGAGCTGAAGGGCATCGACTTCAAAGAGGACGGCAACATCCTGGGCC
ACAAGCTCGAGTACAACTACAACAGCCACAACGTGTACATCATGGCCGACAAGCAGAAAAAC
GGCATCAAAGTGAACTTCAAGATCCGGCACAACATCGAGGACGGCTCTGTGCAGCTGGCCGA
TCACTACCAGCAGAACACACCTATCGGCGACGGACCTGTGCTGCTGCCTGATAACCACTACCT
GAGCTACCAGAGCGCCCTGAGCAAGGACCCTAACGAGAAGAGGGACCACATGGTGCTGCTG
GAATTCGTGACAGCCGCTGGCATCACACTCGGCATGGACGAGCTGTACAAGGGTACCAACCT
GACCATGAGCAACATGACCAGCAGCCACATCTGCTGCAACTTTCTGAACATGTGGAAGAAGT
CCAAGATCTCCACCATGTACTACCTGAACCAGGACGCCAAGCTGAGCAACCTGTTCCTGCAA
GCCAGCTCTCCCACAACCGGCACAGCTCCTAGAAGCCAGAGCAGACTGAGCGTGTGCCCTAG
CACACAGGACATCTGCAGAATCTGCCACTGTGAAGGCGACGAGGAAAGCCCTCTGATCACCC
CTTGCAGATGCACCGGCACACTGAGATTCGTGCACCAGAGCTGTCTGCACCAGTGGATCAAG
AGCAGCGACACCAGATGCTGCGAGCTGTGCAAATACGACTTCATCATGGAAACGAAGCTGAA
GCCCCTGAGGAAGTGGGAGAAGCTGCAGATGACCACCAGCGAGAGAAGAAAGATCTTCTGC
AGCGTGACCTTCCACGTGATCGCCGTGACCTGTGTCGTCTGGTCCCTGTACGTGCTGATCGAC
AGAACCGCCGAAGAGATCAAGCAGGGCGTGCTGGAATGGCCCTTCTGGACAAAACTGGTGG
TGGTGGCCATCGGCTTCACAGGCGGACTGGTGTTTATGTACGTGCAGTGCAAGGTGTACGTCC
AGCTGTGGCGGAGACTGAAGGCCTACAACAGAGTGATCTTCGTGCAGAACTGCCCCGACACC
GCTAACAAGCTGGAAAAGAACTTCCCCTGCAACGTGAACACCGAGATCAAGGACGCTGTGG
TGGTGCCAGTGCCTCAGACCGGATCTAACACACTGCCTACAGCTGAGGGCGCTCCTCCTGAA
GTGATCCCTGTTCCATGTACCTGGGGCAGCGCTAGCTTCAAGAACCTGATCTGGCTGAAAGAG
GTGGACAGCACCCAAGAGCGACTGAAAGAATGGAACGTGTCCTACGGCACAGCCCTGGTGG
CCGACAGACAAACAAAAGGCAGAGGCGGCCTGGGCAGAAAGTGGCTGTCTCAAGAAGGCG
GGCTGTACTTCTCCTTCCTGCTGAACCCCAAAGAGTTCGAGAACCTGCTGCAGCTGCCTCTGG
TTCTGGGCCTGTCTGTGTCTGAAGCCCTGGAAGAGATTACAGAGATCCCCTTCAGCCTGAAGT

Biology Open • Supplementary information

GGCCCAACGACGTGTACTTCCAAGAGAAAAAGGTGTCCGGCGTGCTGTGCGAACTGTCCAA
GGACAAGCTGATCGTCGGCATCGGCATTAACGTGAACCAGAGAGAGATCCCAGAGGAAATCA
AGGACAGAGCCACCACACTGTACGAGATCACAGGCAAGGACTGGGACAGAAAAGAAGTGCT
GCTGAAGGTCCTGAAGCGGATCAGCGAGAATCTGAAGAAGTTCAAAGAGAAGTCCTTTAAA
GAGTTCAAGGGCAAGATCGAGAGCAAGATGCTGTACCTCGGCGAGGAAGTGAAGCTGCTCG
GCGAAGGCAAGATCACCGGAAAGCTCGTGGGCCTGAGCGAAAAAGGCGGCGCTCTGATCCT
GACCGAGGAAGGGATCAAAGAGATCCTGAGCGGCGAGTTCTCCCTGAGAAGAAGCTG*

Fig. S1. Map and nuclotide sequence of eYFP-MARCH1-BioID YFP, MARCH1 and BioID domains of the fusion protein aswell as important restriction sites are mapped. Map colors correspond to highlight color in the nucleotide sequence.

Biology Open • Supplementary information

atgaagaaggccgaaatgggacgattcagtatttccccggatgaagacagcagcagctacagttccaacagcgacttcaactactcctaccccaccaag
caagctgctctgaaaagccattatgcagatgtagatcctgaaaaccagaactttttacttgaatcgaatttggggaagaagaagtatgaaacagaatttcatc
caggtactacttcctttggaatgtcagtatttaatctgagcaatgcgattgtgggcagtggaatccttgggctttcttatgccatggctaatactggaattgctct
ttttataattctcttgacatttgtgtcaatattttccctgtattctgttcatctccttttgaagactgccaatgaaggagggtctttattatatgaacaattgggatataa
ggcatttggattagttggaaagcttgcagcatctggatcaattacaatgcagaacattggagctatgtcaagctacctcttcatagtgaaatatgagttgccttt
ggtgatccaggcattaacgaacattgaagataaaactggattgtggtatctgaacgggaactatttggttctgttggtgtcattggtggtcattcttcctttgtcg
ctgtttagaaatttaggatatttgggatataccagtggcctttccttgttgtgtatggtgttctttctgattgtggtcatttgcaagaaatttcaggttccgtgtcctgt
ggaagctgctttgataattaacgaaacaataaacaccaccttaacacagccaacagctcttgtacctgctttgtcacataacgtgactgaaaatgactcttgc
agacctcactattttattttcaactcacagactgtctatgctgtgccaattctgatcttttcatttgtctgtcatcctgctgttcttcccatctatgaagaactgaaag
accgcagccgtagaagaatgatgaatgtgtccaagatttcatttttgctatgtttctcatgtatctgcttgccgccctctttggatacctaacattttacgaacat
gttgagtcagaattgcttcatacctactcttctatcttgggaactgatattcttcttctcattgtccgtctggctgtgttaatggctgtgaccctgacagtaccagta
gttattttcccaatccggagttctgtaactcacttgttgtgtgcatcaaaagatttcagttggtggcgtcatagtctcattacagtgtctatcttggcatttaccaat
ttacttgtcatctttgtcccaactattagggatatctttggttttattggtgcatctgcagcttctatgttgatttttattcttccttctgccttctatatcaagttggtgaa
gaaagaacctatgaaatctgtacaaaagattggggctttgttcttcctgttaagtggtgtactggtgatgaccggaagcatggccttgattgttttggattgggt
acacaatgcacctggaggtggccatGACTACAAGGACGACGACGACAAG

Fig. S2. Nucleotide sequence of SNAT2-FLAG. Lowercase letters constitute the sequence of SNAT2 while uppercase letters represent the sequence of the FLAG-TAG.

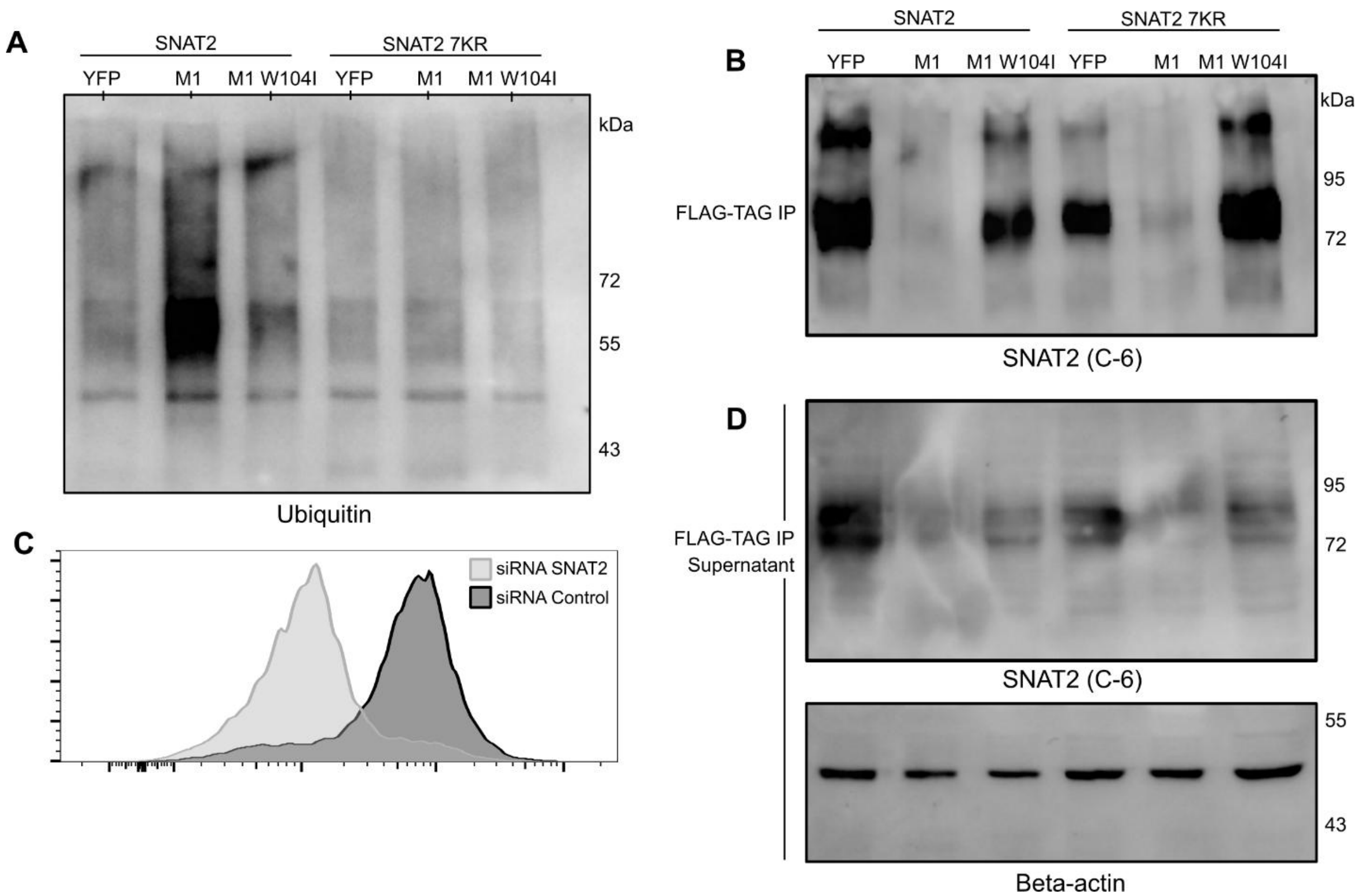

Fig. S3. SNAT2 is ubiquitinated by MARCH1

- **A.** A repeat of the experiment detecting ubiquitinated SNAT2 in cells transfected with MARCH1 presented in Figure 5. This new western blot includes the SNAT2 7KR mutant and was performed in denaturaing RIPA buffer.
- **B.** Western blot showing SNAT2 recovered by FLAG-TAG IP in SNAT2-FLAG + M1 transfected cells.
- **C.** Confirmation of the specificity of the SNAT2 (C-6) antibody by siRNA knockdown.
- **D.** Detection of SNAT2 and loading control from the FLAG-TAG IP supernatant.

Biology Open • Supplementary information

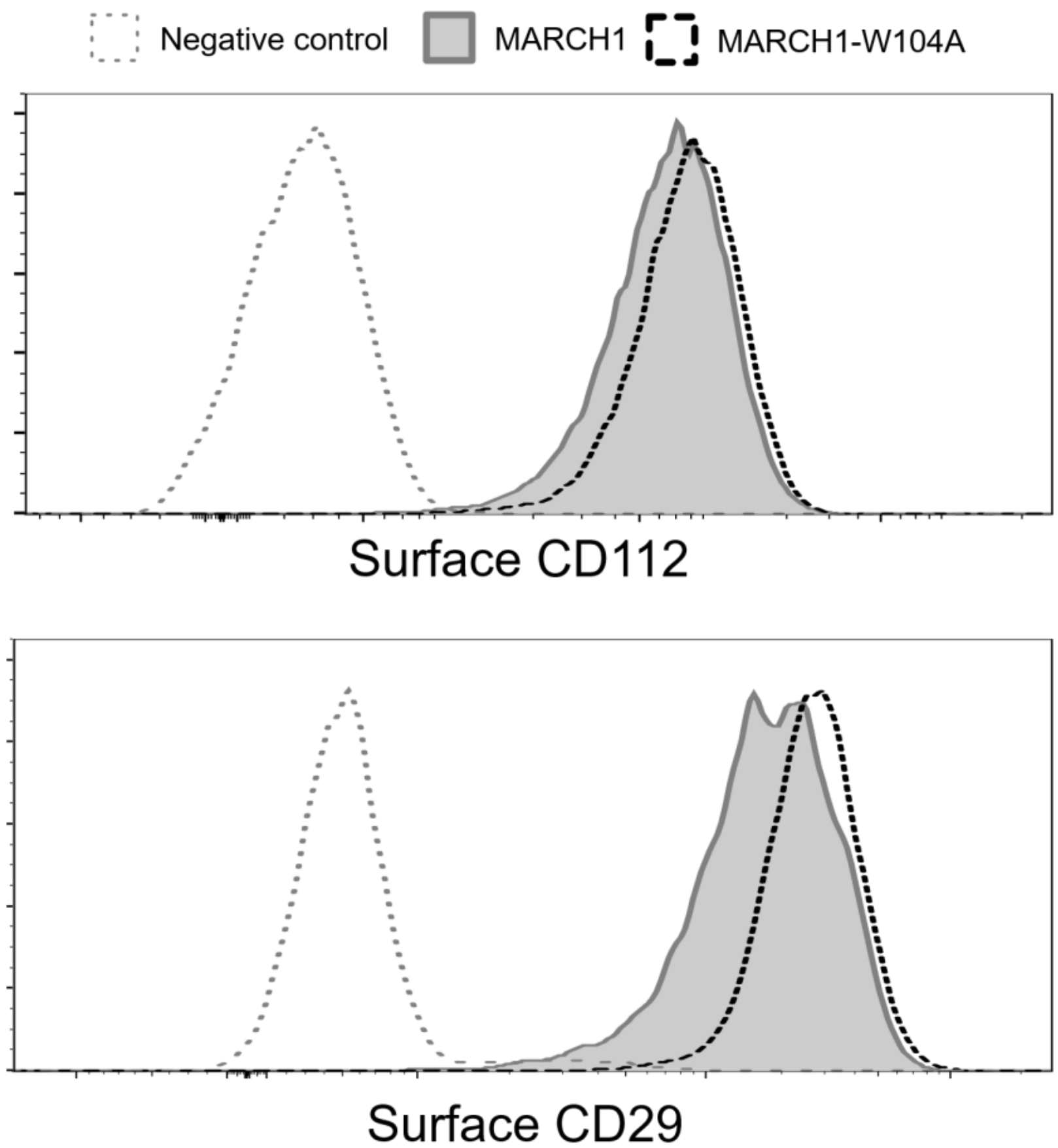

Fig. S4. The presence of MARCH1 but not MARCH1W104A has an effect on the surface expression of CD29 and CD112. HEK293 cells were transfected with either MARCH1 or MARCH1W104A. Levels of surface endogenous CD29 and CD112 were measured by flow-cytometry after 48 hours. Negative controls consist of a secondary antibody staining alone (upper panel), or an irrelevant I-A-specific antibody plus secondary antibody (lower panel).

Table S1. Conformation and known membrane association of proteins identified in MARCH1 BioID experiments.

Protein	Trans membrane	Plasma membrane association	Cytoplasmic lysine residue	References	Notes
Basigin **BSG (CD147)**	Yes	Yes	Yes	(Kanekura, Chen and Kanzaki, 2002)	
Synaptosomal-associated protein 23 **SNAP23**	No	Yes	Yes	(Hatsuzawa and Sakurai, 2020; Naskar and Puri, 2017)	
Solute carrier family 38, member 2 **SNAT2 (SLC38A2)**	Yes	Yes	Yes	(Zhang, Zander and Grewer, 2011)	
Nectin-2 **NECTIN2 (CD112)**	Yes	Yes	Yes	(Samanta et al., 2012)	
Integrin beta-1 **ITGB1 (CD29)**	Yes	Yes	Yes	(Zuidema et al., 2022)	
Transferrin receptor **TFRC (CD71)**	Yes	Yes	Yes	(Jabara et al., 2016)	
Solute carrier family 12 member 2 **NKCC1 (SLC12A2)**	Yes	Yes	Yes	(Payne et al., 1995; Yang, Wang and Cao, 2020)	
4F2 cell-surface antigen heavy chain **SLC3A2 (CD98hc)**	Yes	Yes	Yes	(Quackenbush et al., 1987)	
Non-specific serine/threonine protein kinase **MARK3**	No	Yes	Yes	(Moravcevic et al., 2010)	Associated to phospholipids
Myristoylated alanine-rich C-kinase substrate **MARCKS**	No	Yes	Yes	(Carballo et al., 1999; Issara-Amphorn et al., 2023; Thelen et al., 1991)	Associated to PKC
Occludin **OCLN**	Yes	Yes	Yes	(Elias et al., 2009)	
Adenosyl homocysteinase 3 **AHCYL2 (Long-IRBIT)**	No	Possible	Yes	(Ando, Mizutani and Mikoshiba, 2009)	(Uniprot) Note: Associates with membranes when phosphorylated, probably through interaction with ITPR1.
Anion exchange protein 2 **AE2 (SLC4A2)**	Yes	Yes	Yes	(Aranda et al., 2004; Itoh et al., 2021)	Associates with long-IRBIT for lysosomal degradation.
Feline leukemia virus subgroup C receptor-related protein 1 **FLVCR1**	Yes	Yes	Yes	(Fiorito and Tolosano, 2024; Quigley et al., 2004; Ri et al., 2024)	Associated to heme and choline transport.
Carboxypeptidase D **CPD**	Yes	Yes	Yes	(Kuroki et al., 1995)	
Leucyl-cystinyl aminopeptidase **LNPEP**	Yes	Yes	Yes	(Rogi et al., 1996)	(Uniprot) In brain only the membrane-bound form is found. The protein resides in intracellular vesicles

Biology Open • Supplementary information

					together with GLUT4 and can then translocate to the cell surface in response to insulin and/or oxytocin.
Spectrin beta chain, non-erythrocytic 2 **SPTBN2**	No	Possible	Yes	(Deng et al., 2024)	SPTBN2 suppresses ferroptosis in NSCLC cells by facilitating SLC7A11 membrane trafficking and localization
Solute carrier family 38, member 1 **SNAT1 (SLC38A1)**	Yes	Yes	Yes	(Melone et al., 2004)	

Table S2.

Available for download at
https://journals.biologists.com/bio/article-lookup/doi/10.1242/bio.061700#supplementary-data

Biology Open • Supplementary information

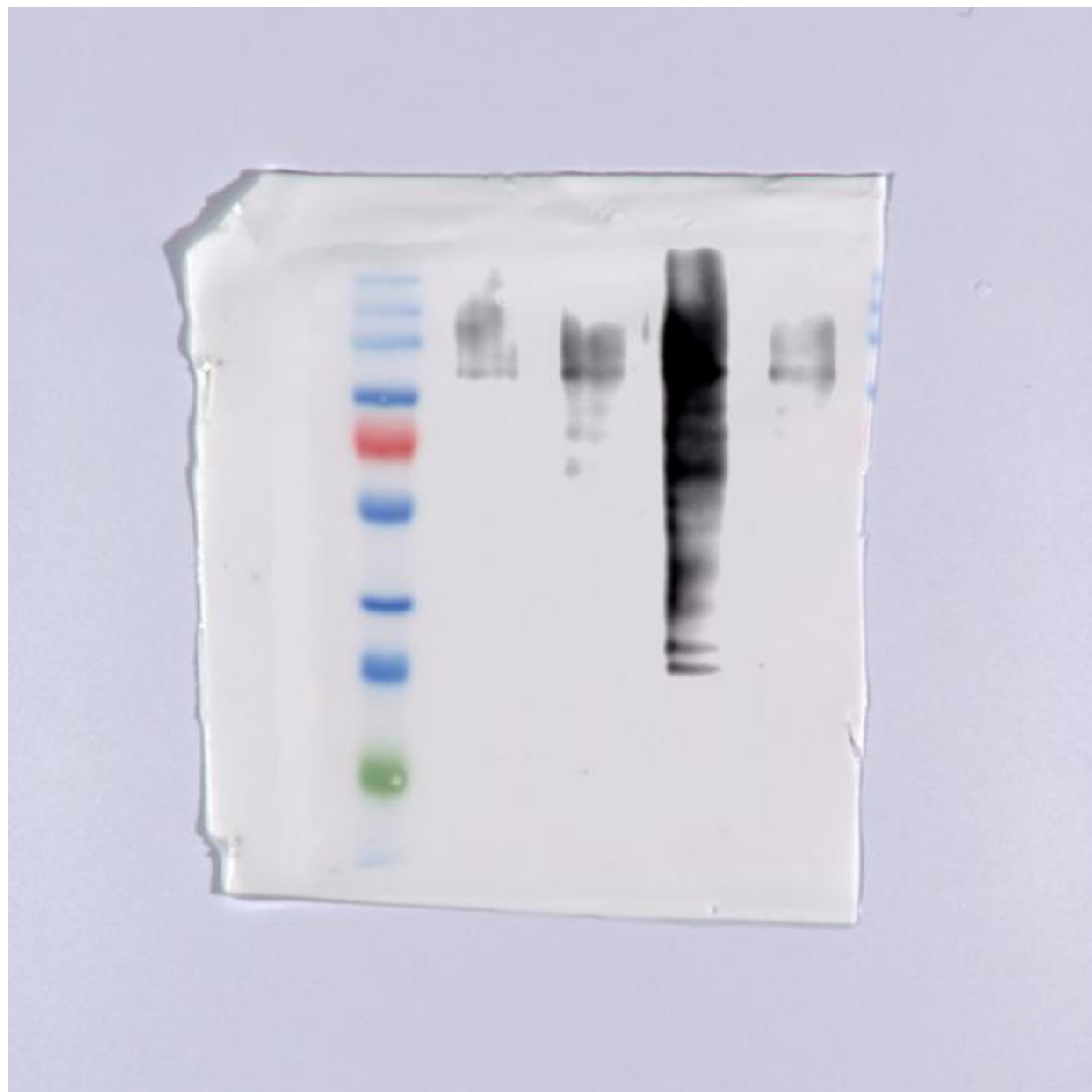

Fig. S5. Blot transparency related to figure 5C

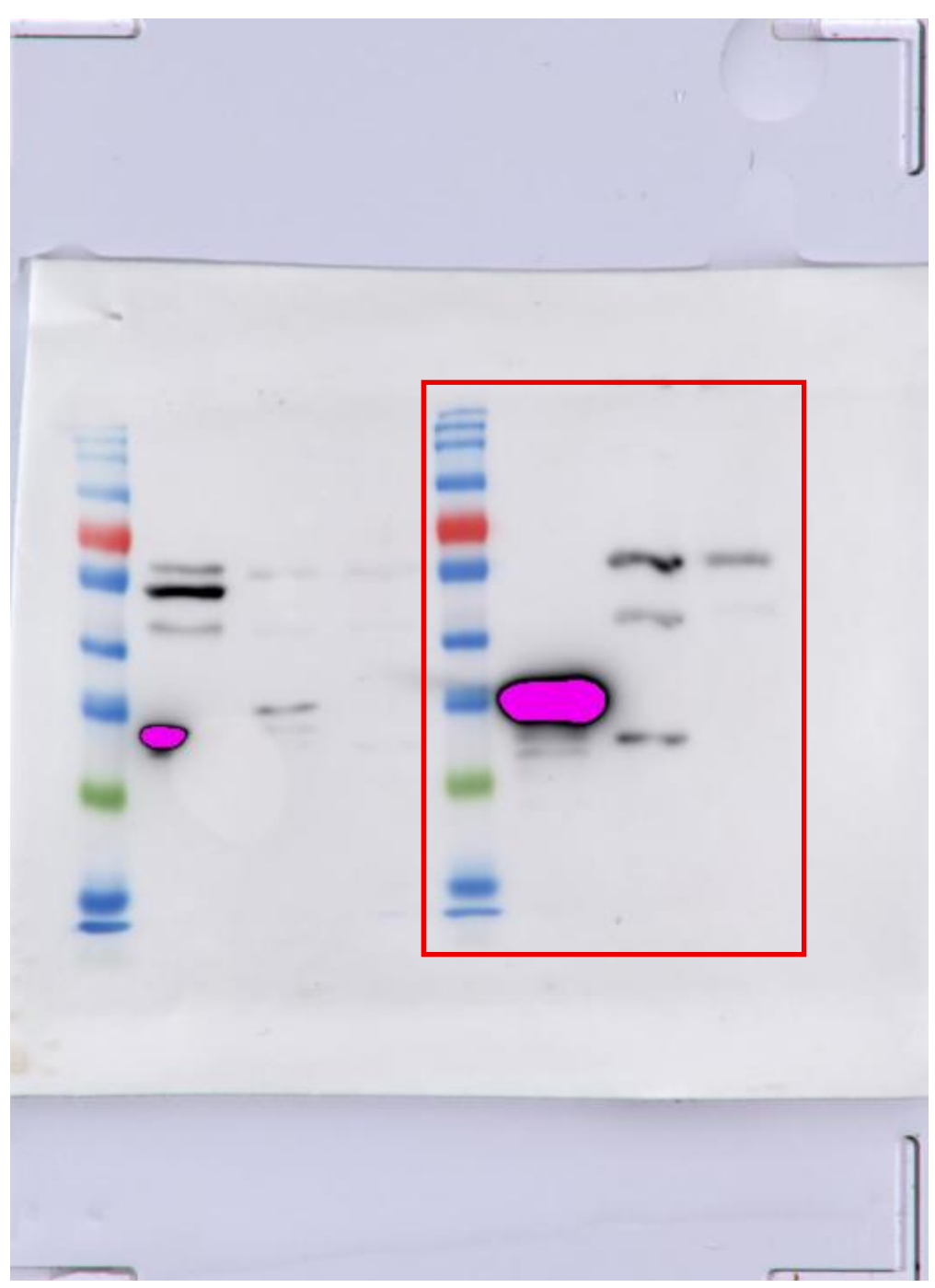

Fig. S6. Blot transparency related to figure 1D

Biology Open • Supplementary information

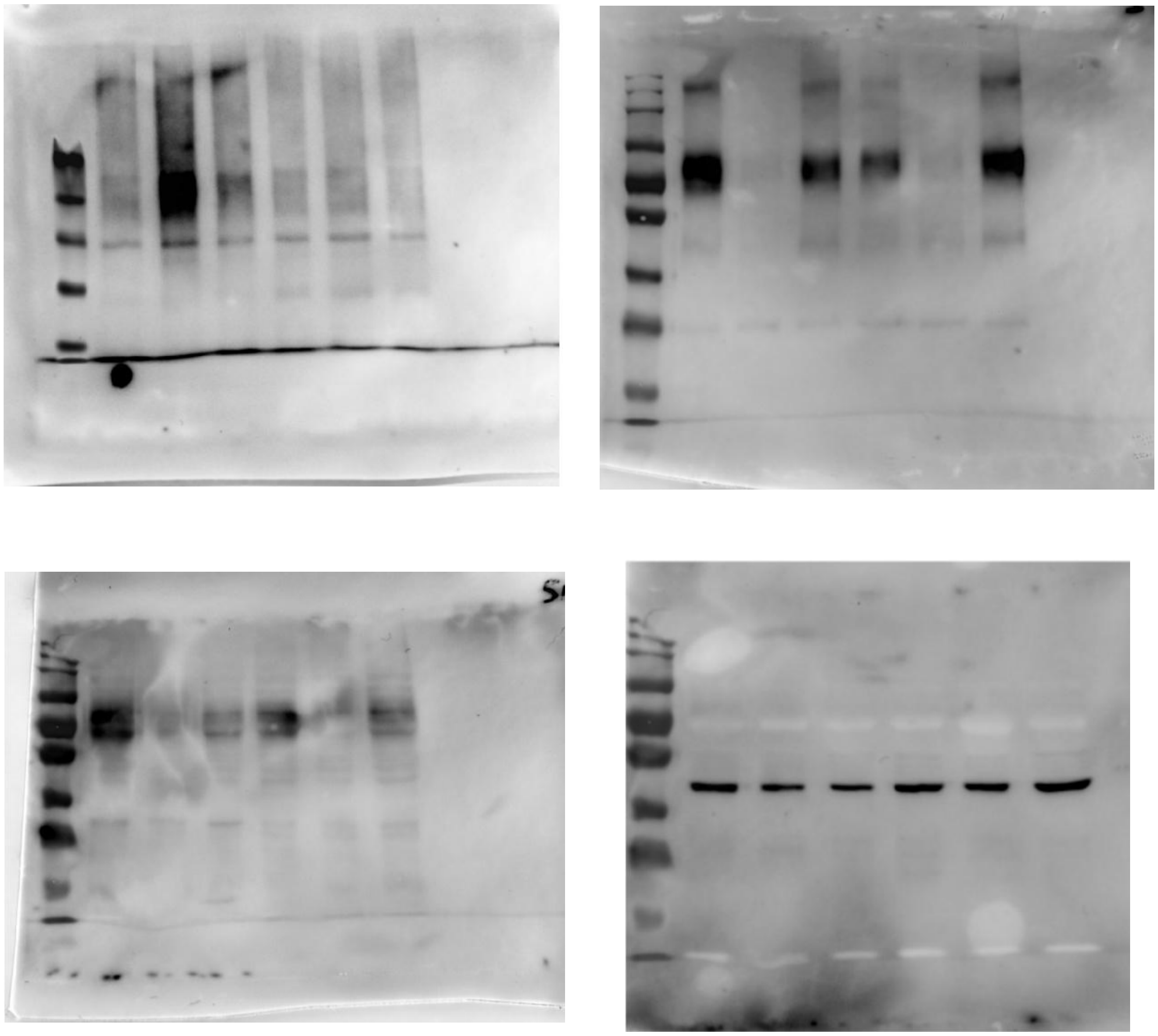

Fig. S7. Blot transparency related to figure S3

Refererences

Ando, H., Mizutani, A. and Mikoshiba, K. (2009). An IRBIT homologue lacks binding activity to inositol 1,4,5-trisphosphate receptor due to the unique N-terminal appendage. *J Neurochem* **109**, 539-50.

Aranda, V., Martinez, I., Melero, S., Lecanda, J., Banales, J. M., Prieto, J. and Medina, J. F. (2004). Shared apical sorting of anion exchanger isoforms AE2a, AE2b1, and AE2b2 in primary hepatocytes. *Biochem Biophys Res Commun* **319**, 1040-6.

Carballo, E., Pitterle, D. M., Stumpo, D. J., Sperling, R. T. and Blackshear, P. J. (1999). Phagocytic and macropinocytic activity in MARCKS-deficient macrophages and fibroblasts. *Am J Physiol* **277**, C163-73.

Deng, J., Lin, X., Qin, J., Li, Q., Zhang, Y., Zhang, Q., Ji, C., Shen, S., Li, Y., Zhang, B. and Lin, N. (2024). SPTBN2 suppresses ferroptosis in NSCLC cells by facilitating SLC7A11 membrane trafficking and localization. *Redox Biol* **70**, 103039.

Elias, B. C., Suzuki, T., Seth, A., Giorgianni, F., Kale, G., Shen, L., Turner, J. R., Naren, A., Desiderio, D. M. and Rao, R. (2009). Phosphorylation of Tyr-398 and Tyr-402 in occludin prevents its interaction with ZO-1 and destabilizes its assembly at the tight junctions. *J Biol Chem* **284**, 1559-69.

Fiorito, V. and Tolosano, E. (2024). Unearthing FLVCR1a: tracing the path to a vital cellular transporter. *Cell Mol Life Sci* **81**, 166.

Hatsuzawa, K. and Sakurai, C. (2020). Regulatory Mechanism of SNAP23 in Phagosome Formation and Maturation. *Yonago Acta Medica* **63**, 135-145.

Issara-Amphorn, J., Sjoelund, V. H., Smelkinson, M., Montalvo, S., Yoon, S. H., Manes, N. P. and Nita-Lazar, A. (2023). Myristoylated, alanine-rich C-kinase substrate (MARCKS) regulates toll-like receptor 4 signaling in macrophages. *Scientific Reports* **13**, 19562.

Itoh, R., Hatano, N., Murakami, M., Mitsumori, K., Kawasaki, S., Wakagi, T., Kanzaki, Y., Kojima, H., Kawaai, K., Mikoshiba, K. et al. (2021). Both IRBIT and long-IRBIT bind to and coordinately regulate Cl(-)/HCO(3)(-) exchanger AE2 activity through modulating the lysosomal degradation of AE2. *Scientific Reports* **11**, 5990.

Jabara, H. H., Boyden, S. E., Chou, J., Ramesh, N., Massaad, M. J., Benson, H., Bainter, W., Fraulino, D., Rahimov, F., Sieff, C. et al. (2016). A missense mutation in TFRC, encoding transferrin receptor 1, causes combined immunodeficiency. *Nat Genet* **48**, 74-8.

Kanekura, T., Chen, X. and Kanzaki, T. (2002). Basigin (CD147) is expressed on melanoma cells and induces tumor cell invasion by stimulating production of matrix metalloproteinases by fibroblasts. *Int J Cancer* **99**, 520-8.

Kuroki, K., Eng, F., Ishikawa, T., Turck, C., Harada, F. and Ganem, D. (1995). gp180, a host cell glycoprotein that binds duck hepatitis B virus particles, is encoded by a member of the carboxypeptidase gene family. *J Biol Chem* **270**, 15022-8.

Melone, M., Quagliano, F., Barbaresi, P., Varoqui, H., Erickson, J. D. and Conti, F. (2004). Localization of the glutamine transporter SNAT1 in rat cerebral cortex and neighboring structures, with a note on its localization in human cortex. *Cereb Cortex* **14**, 562-74.

Moravcevic, K., Mendrola, J. M., Schmitz, K. R., Wang, Y. H., Slochower, D., Janmey, P. A. and Lemmon, M. A. (2010). Kinase associated-1 domains drive MARK/PAR1 kinases to membrane targets by binding acidic phospholipids. *Cell* **143**, 966-77.

Naskar, P. and Puri, N. (2017). Phosphorylation of SNAP-23 regulates its dynamic membrane association during mast cell exocytosis. *Biol Open* **6**, 1257-1269.

Payne, J. A., Xu, J. C., Haas, M., Lytle, C. Y., Ward, D. and Forbush, B., 3rd. (1995). Primary structure, functional expression, and chromosomal localization of the bumetanide-sensitive Na-K-Cl cotransporter in human colon. *J Biol Chem* **270**, 17977-85.

Quackenbush, E., Clabby, M., Gottesdiener, K. M., Barbosa, J., Jones, N. H., Strominger, J. L., Speck, S. and Leiden, J. M. (1987). Molecular cloning of complementary DNAs encoding the heavy chain of the human 4F2 cell-surface antigen: a type II membrane glycoprotein involved in normal and neoplastic cell growth. *Proceedings of the National Academy of Sciences* **84**, 6526-6530.

Biology Open • Supplementary information

Quigley, J. G., Yang, Z., Worthington, M. T., Phillips, J. D., Sabo, K. M., Sabath, D. E., Berg, C. L., Sassa, S., Wood, B. L. and Abkowitz, J. L. (2004). Identification of a human heme exporter that is essential for erythropoiesis. *Cell* **118**, 757-66.

Ri, K., Weng, T. H., Claveras Cabezudo, A., Josting, W., Zhang, Y., Bazzone, A., Leong, N. C. P., Welsch, S., Doty, R. T., Gursu, G. et al. (2024). Molecular mechanism of choline and ethanolamine transport in humans. *Nature* **630**, 501-508.

Rogi, T., Tsujimoto, M., Nakazato, H., Mizutani, S. and Tomoda, Y. (1996). Human placental leucine aminopeptidase/oxytocinase. A new member of type II membrane-spanning zinc metallopeptidase family. *J Biol Chem* **271**, 56-61.

Samanta, D., Ramagopal, U. A., Rubinstein, R., Vigdorovich, V., Nathenson, S. G. and Almo, S. C. (2012). Structure of Nectin-2 reveals determinants of homophilic and heterophilic interactions that control cell-cell adhesion. *Proc Natl Acad Sci U S A* **109**, 14836-40.

Thelen, M., Rosen, A., Nairn, A. C. and Aderem, A. (1991). Regulation by phosphorylation of reversible association of a myristoylated protein kinase C substrate with the plasma membrane. *Nature* **351**, 320-322.

Yang, X., Wang, Q. and Cao, E. (2020). Structure of the human cation-chloride cotransporter NKCC1 determined by single-particle electron cryo-microscopy. *Nat Commun* **11**, 1016.

Zhang, Z., Zander, C. B. and Grewer, C. (2011). The C-terminal domain of the neutral amino acid transporter SNAT2 regulates transport activity through voltage-dependent processes. *Biochem J* **434**, 287-96.

Zuidema, A., Atherton, P., Kreft, M., Hoekman, L., Bleijerveld, O. B., Nagaraj, N., Chen, N., Fassler, R. and Sonnenberg, A. (2022). PEAK1 Y635 phosphorylation regulates cell migration through association with Tensin3 and integrins. *J Cell Biol* **221**.
